# The geographical distribution of the family-genetic risk score for drug use disorder in Sweden and its co-localization with areas of social deprivation

**DOI:** 10.1017/S0033291724002745

**Published:** 2024-11

**Authors:** Kenneth S. Kendler, Ali Mansourian, Pengxiang Zhao, Henrik Ohlsson, Kathleen Stewart, Jan Sundquist, Bo Malmberg, Kristina Sundquist

**Affiliations:** 1 Virginia Institute for Psychiatric and Behavioral Genetics, Virginia Commonwealth University, Richmond, VA, USA; 2 Department of Psychiatry, Virginia Commonwealth University, Richmond, VA, USA; 3 Department of Physical Geography and Ecosystem Science, Lund University, Malmö, Sweden; 4 Center for Primary Health Care Research, Lund University, Malmö, Sweden; 5 Center for Geospatial Information System, Department of Geographical Sciences, University of Maryland, College Park, MD 20742, USA; 6 Department of Family Medicine and Community Health, Department of Population Health Science and Policy, Icahn School of Medicine at Mount Sinai, New York, NY, USA; 7 Department of Geography of Stockholm University, Stockholm, Sweden

**Keywords:** drug use disorder, genetic risk, geography, social deprivation, Sweden

## Abstract

**Background:**

Drug use Disorder (DUD), the risk for which is substantially influenced by both genetic and social factors, is geographically concentrated in high-risk regions. An important step toward understanding this pattern is to examine geographical distributions of the genetic liability to DUD and a key demographic risk factor – social deprivation.

**Methods:**

We calculated the mean family genetic risk score (FGRS) for DUD ((FGRS_DUD_) and social deprivation for each of the 5983 areas Demographic Statistical Areas (DeSO) for all of Sweden and used geospatial techniques to analyze and map these factors.

**Results:**

Using 2018 data, substantial spatial heterogeneity was seen in the distribution of the genetic risk for DUD in Sweden as a whole and in its three major urban centers which was confirmed by hot-spot analyses. Across DeSOs, FGRS_DUD_ and s.d. levels were substantially but imperfectly correlated (*r* = + 0.63), with more scattering at higher FGRS_DUD_ and s.d. scores. Joint mapping across DeSOs for FGRS_DUD_ and s.d. revealed a diversity of patterns across Sweden. The stability of the distributions of FGRS_DUD_ and s.d. in DeSOs within Sweden over the years 2012–2018 was quite high.

**Conclusions:**

The geographical distribution of the genetic risk to DUD is quite variable in Sweden. DeSO levels of s.d. and FRGS_DUD_ were substantially correlated but also disassociated in a number of regions. The observed patterns were largely consistent with known trends in the human geography of Sweden. This effort lays the groundwork for further studies of the sources of geographic variation in rates of DUD.

Drug Use Disorder (DUD), a highly multifactorial syndrome with major contributions from biological, psychological, and social factors(Blanco et al., 2013; Kendler, Ohlsson, Edwards, Sundquist, & Sundquist, 2017; West, 2006), is often geographically and temporally concentrated (Brownstein, Green, Cassidy, & Butler, 2010; Faris & Dunham, 1939; Linton, Jennings, Latkin, Gomez, & Mehta, 2014; Petronis & Anthony, 2003; Stewart et al., 2023; Thomas, Richardson, & Cheung, 2008). A long tradition of epidemiological studies has shown that risk for both illicit substance use and DUD are associated with low levels of education, unemployment, low income and residence in areas of high social deprivation (Faris & Dunham, 1939; Grant et al., 2009; Hawkins, Catalano, & Miller, 1992; Kendler et al., 2017; Warner, Kessler, Hughes, Anthony, & Nelson, 1995). With the widening availability of familial-genetic data in human populations, it has become possible to map genetic risk factors for traits or disorders some of which show spatial heterogeneity (Abdellaoui et al., 2019; Fan et al., 2018; Kerminen et al., 2019; Sohail, Izarraras-Gomez, & Ortega-Del Vecchyo, 2021). A substantial genetic-epidemiological and molecular genetic literature now supports the important role of genetic risk factors in the etiology of DUD (Deak & Johnson, 2021), with heritability estimates from twin studies typically in the range of 50% (Kendler, Rosmalen, Ohlsson, Sundquist, & Sundquist, 2023e; Tsuang, Bar, Harley, & Lyons, 2001).

In this paper, we utilize the family-genetic risk score (FGRS), a quantitative measure of genetic risk based on the rates of a particular disorder in 1st through 5th degree relatives adjusted for age, sex, geographical area, year of birth and familial environmental effects. This FGRS has performed well in a number of empirical applications with simulations suggesting that it provides a good and relatively unbiased measure of genetic risk (Kendler et al., 2023a, 2023b, 2023e; Kendler, O., Sundquist, & Sundquist, 2023c; Kendler, Ohlsson, Sundquist, & Sundquist, 2023d; Kendler, Ohlsson, Sundquist, & Sundquist, 2021a; Kendler, Ohlsson, Sundquist, & Sundquist, 2021b). Using the FGRS for DUD (FGRS_DUD_), we address the following three questions:

1. What is the geographical distribution of the FGRS_DUD_ in the country of Sweden and its three major urban centers – Stockholm, Gothenburg, and Malmö – and to what degree does FGRS_DUD_ display significant spatial heterogeneity?
2. What is the joint geographical distribution of FGRS_DUD_ and social deprivation (s.d.)? That is, to what degree do these two major risk factors for DUD co-localize within regions of Sweden?
3. What is the temporal stability of the spatial distribution of FGRS_DUD_ alone and the joint distributions of FGRS_DUD_ and s.d.?

## Methods

We collected information on individuals from Swedish population-based registers with national coverage linking each person's unique personal identification number which, to preserve confidentiality, was replaced with a pseudonymized serial number by Statistics Sweden. We secured ethical approval for this study from the Regional Ethical Review Board in Lund (No. 2008/409 and later amendments). In the database, we included for each individual a family genetic risk score (FGRS_DUD_) for Drug Use Disorder. For our definition of DUD, see Appendix Table 1. The FGRS is calculated from morbidity risks for disorders in first-degree through fifth-degree relatives, controlling for cohabitation effects, and thus arise from phenotypes in extended pedigrees, not from molecular genetic data. See Appendix Table 2 for more details. In the database, we also included, for the years 2012, 2015, and 2018, the DeSO area where the individual resided. The DeSO areas divide Sweden into 5983 regions which have between 700 and 2700 inhabitants. The division takes into account the geographical conditions so that the boundaries commonly follow streets, waterways and railways. Important building blocks used to create DeSOs are urban areas and electoral districts. For each of the DeSO areas we created a neighborhood social deprivation (s.d.) index based on register data for all residents in the neighborhood aged 25–64. We used deprivation indicators employed by past studies to characterize neighborhood environments and then used a principal component analysis to calculate a z-score (Winkleby, Sundquist, & Cubbin, 2007). The following four variables were included: low educational status (defined as less than 10 years of formal education); low income (from all sources, including that from interest and dividends, which was defined as less than 50% of individual median income); unemployment (defined as not employed; excluding full-time students, those completing compulsory military service, and early retirees); and social welfare assistance.

For each DeSO area and year, we calculated the mean FGRS_DUD_ for all individuals aged 15 to 35, peak years for DUD, who were born in Sweden to Swedish born parents. To account for differences in population size across areas, we used the predicted values from a multilevel model with individuals nested within DeSO areas in which we controlled for year of birth and sex. This means that areas with fewer individuals and larger variation within the area are shrunken towards the overall mean value. To uncover patterns of FGRS at the DeSO-level, a spatial clustering analysis was implemented to detect hot- and coldspots of FGRS using the Getis-Ord Gi*statistic (Getis & Ord, 1992). The G* statistic has been commonly used in spatial statistics to quantify spatial clustering in a dataset (Amune & Amilian, 2023; Luenam & Puttanapong, 2019; Tsai, Lin, Chu, & Perng, 2009).

Hotspots are identified as groups of DeSOs where high values cluster, resulting in higher G* scores. Conversely, coldspots are where low values cluster, leading to lower G* scores. In this study, the calculation of the G* statistic involved comparing the FGRS values of a DeSO and its neighboring DeSOs to the expected value from the whole dataset under the assumption of spatial randomness. The basic statistic can be calculated as:
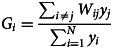
where *W*_*ij*_ is the spatial weight between *ith* and *jth* DeSOs; *y*_*j*_ is the FGRS at DeSO *j*; and *N* is the number of DeSOs. Developing the spatial weights *W*_*ij*_ is the first step to calculate G* statistic. In this study, adjacency was defined using a first order queen polygon continuity, which is constructed based on the DeSOs that share common boundaries and vertices. If two DeSOs are adjacent, the spatial weight is set as 1; otherwise, it is set as 0.

The *z* scores and *p* values resulting from the spatial statistical analysis indicate the spatial clustering of DeSOs with either high or low FGRS values. Note that a DeSO with a high FGRS value may not be a statistically significant hotspot. To be a statistically significant hotspot, a DeSO will have to have a high FGRS value and be surrounded by other DeSOs with high FGRS values. In this study, the hot- and coldspots were visualized at 90, 95, and 99% significance levels. To detect patterns of FGRS and s.d., we used spatial co-localization analysis. Such co-localization analysis uses bivariate color schemes to compare values. Each variable is categorized, and each category is associated with a specific color. In this study, the FGRS variable is divided into three categories using a quantile method, while the s.d. composite is categorized, based on previous studies(Winkleby et al., 2007), into three groups: below one standard deviation (s.d.) from the mean (low deprivation), above one s.d. from the mean (high deprivation), and within one s.d. of the mean (moderate deprivation). The final bivariate choropleth map is produced based on the two variables with three discrete categories each, providing a total of nine categories for mapping.

## Results

**Figure 1.**
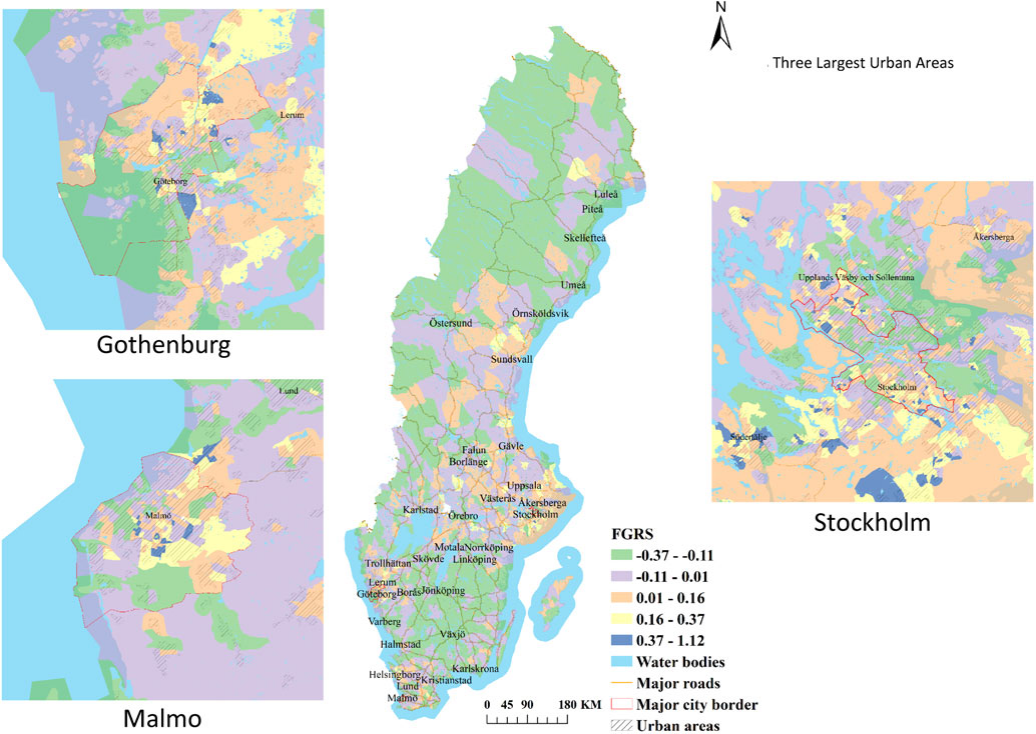
The Mean FGRS_DUD_ by DeSO in 2018 in the Country of Sweden and Its Three Largest Urban Areas – Stockholm, Gothenberg, and Malmö. For each DeSO, we take the mean for all individuals aged 15 to 35 who were born in Sweden to Swedish born parents. The mean FGRS_DUD_ is by definition, 0.00 and for this figure is divided into 5 levels, very low – green, low – purple, intermediate – orange, high – yellow and very high – blue. The national map also contains bodies of water and major roads and names of a number of prominent cities. The insert maps for the three largest cities, also indicates, by hashing, the areas of urbanization.

Figure 1 presents means in 2018 for each DeSO region in Sweden for ages 15–35, classified using natural breaks (with darker blue signifying higher mean FGRS_DUD_) with maps depicting Sweden as well as its three major cities: Stockholm, Malmö, and Gothenburg. Considerable heterogeneity is seen in the distribution of the genetic risk for DUD both for the country as a whole and in its three major urban centers. Each of the major urban areas contain DESOs with relatively high and low levels of mean genetic risk for DUD. Counting as high and low those in the upper two and lower two categories depicted in Fig. 1, Stockholm, which contained 544 DeSOs, had 24.9% with high and 55.9% with low mean levels of FGRS_DUD_. Parallel results for Gothenburg (306 DeSOs) were 29.1 and 51.3% and for Malmö (191 DeSOs) 26.2 and 47.1% (see Appendix Table 3 for full details).

**Figure 2.**
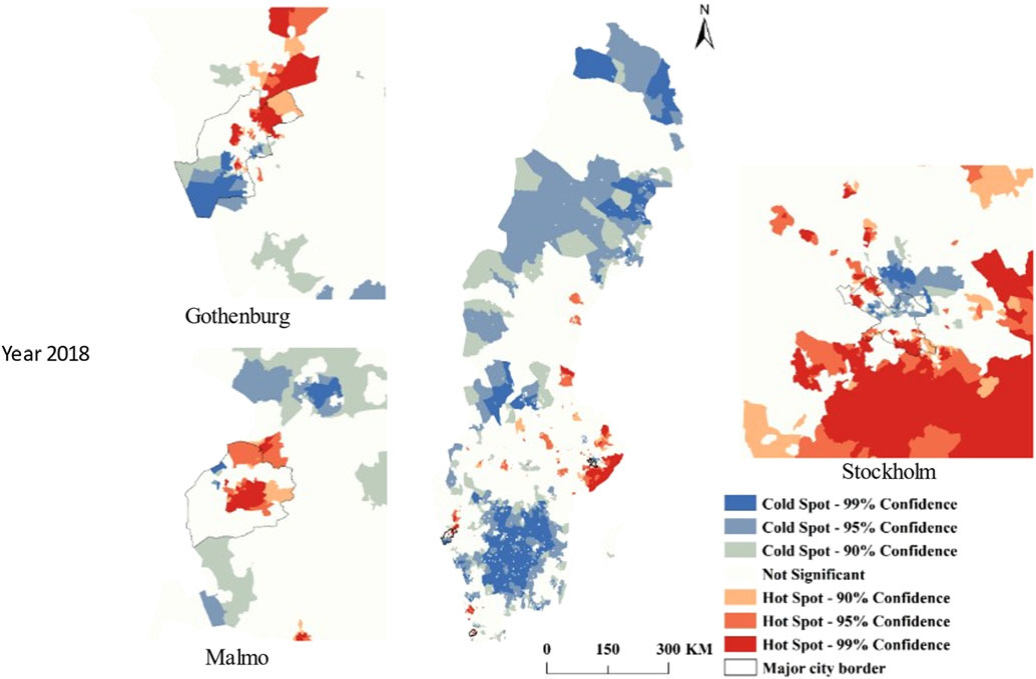
Hot and Cold Spot Analysis of the Mean FGRS_DUD_ By Deso in 2018 in the Country of Sweden and Its Three Largest Urban Areas – Stockholm, Gothenberg, and Malmö. We divide each DeSO into 7 possible categories by Hot spot analysis. White indicated no significant deviation in the mean FGRS_DUD_ from population expectation. Light orange, dark orange and red indicate that the DeSO represents a hot spot with a higher than expected rate of FGRS_DUD_ in the DeSO with, respectively, 90, 95 and 99% confidence. Grey, light blue and dark blue indicate that the DeSO represents a cold spot with a lower than expected rate of FGRS_DUD_ in the DeSO with, respectively, 90, 95 and 99% confidence.

We formally test for spatial autocorrelation of the mean DESO FGRS_DUD_ for 2018 in a hot-spot analysis presented in Fig. 2. Country wide, the largest hot spot is detected in the coastal region east and south of Stockholm while the largest cold spot is in the less densely populated forested areas of southern Sweden made up of the counties of Kronoberg, Jönköping, Östergötland, and Västra Götaland. Hotspots are detected in each of the three cities where 61 DeSOs in Malmö (31.9% of Malmö DeSOs), 89 DeSOs (29.1%) in Gothenberg and 113 DeSOs (20.8%) in Stockholm corresponded to hotspots. The hotspots in these metropolitan regions generally occurred in DeSOs peripheral to parts of the inner city.

**Figure 3.**
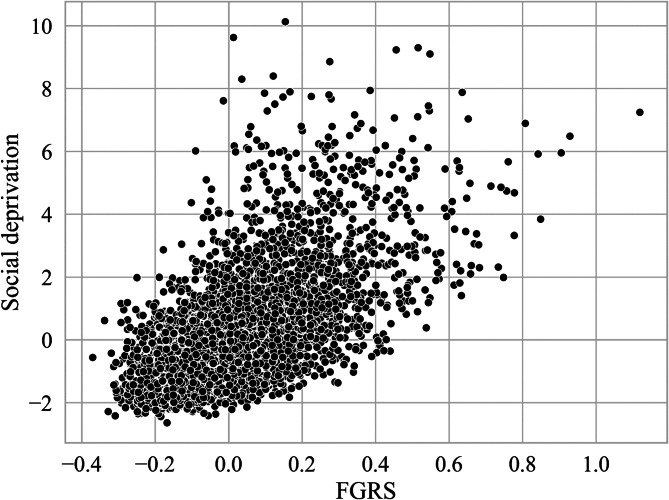
A Scatter-Plot from all DESOs in Sweden of the mean FGRS_DUD_ on the *X*-Axis and the mean Social Deprivation Score on the *Y*-axis. The Pearson correlation between these two variables equals +0.63).

Figure 3 presents a scatter-plot for all DeSO regions for the mean FGRS_DUD_ and the s.d. score in 2018. A considerable but far from perfect correlation is seen in these two measures as assessed at the DeSO level, consistent with the Pearson correlation value of +0.63, with more scattering at higher FGRS_DUD_ and s.d. scores.

**Figure 4.**
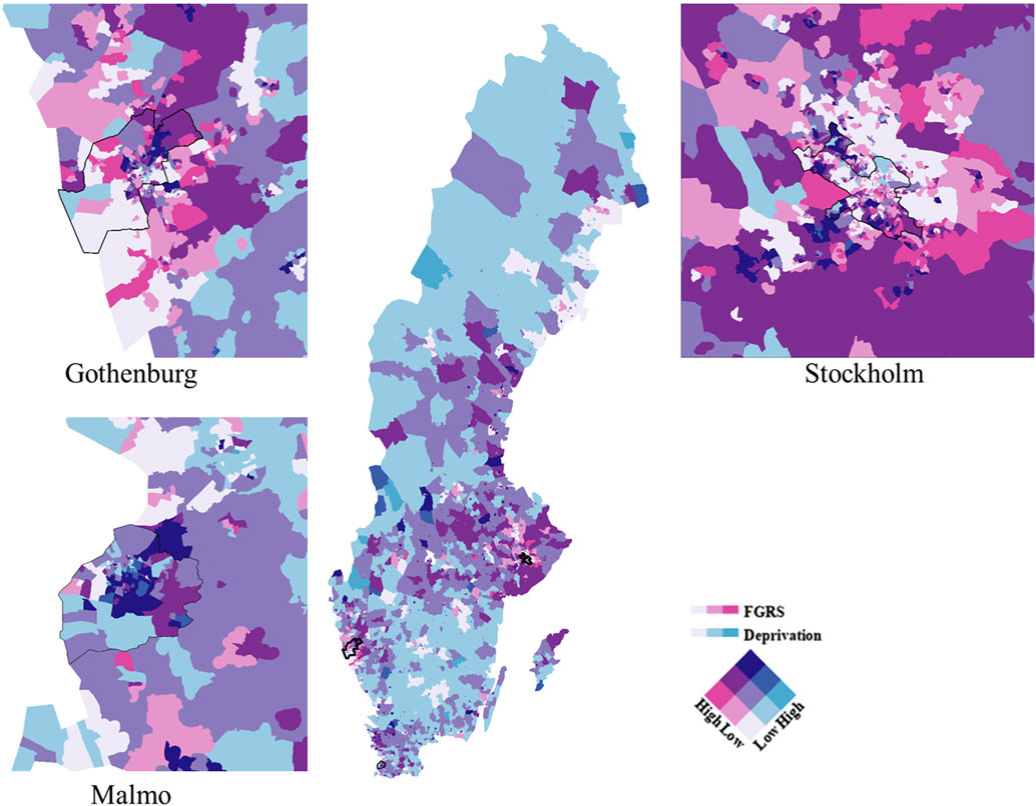
Mean FGRS_DUD_ and Social Deprivation By DeSO in 2018 in the Country of Sweden and Its Three Largest Urban Areas. Each DeSO is assigned to one of 9 possible categories as a function of its Mean FGRS_DUD_ and Social Deprivation score divided into 3 categories of low, medium, and high. The FGRS score is indexes by the shade of red and the level of Deprivation by the shade of blue. So as seen by the color array in the right lower section of the figure, areas of high FGRS_DUD_ and Social Deprivation are marked by dark purple. DeSOs with high FGRS_DUD_ and low Social Deprivation and low FGRS_DUD_ and high Social Deprivation are colored dark red and dark blue respectively.

We examine the joint spatial distribution of the FGRS_DUD_ and the s.d. scores nationwide as well as in the three major metropolitan areas in Fig. 4 where levels of FGRS_DUD_ and the s.d. are divided into lower, intermediate, and higher levels using natural breaks classification. The largest proportion of DESOs in Sweden (light blue) correspond to intermediate levels of s.d. and low levels of FGRS_DUD_.

We also see, most clearly in three cities, that DESOs that represent higher levels of FGRS_DUD_ are associated with low levels of s.d. (bright pink). Malmö contained 33.5% of DeSOs with higher levels of both s.d. and FGRS_DUD_ (depicted in dark purple) with fewer similar areas in Gothenburg (23.5%) and Stockholm (14.9%). Nationwide, there is a prominent area around Stockholm in the east and stretching much but not all the way to the west where most of the DESOs display intermediate levels of s.d. and either intermediate or high levels of FGRS_DUD._

**Figure 5.**
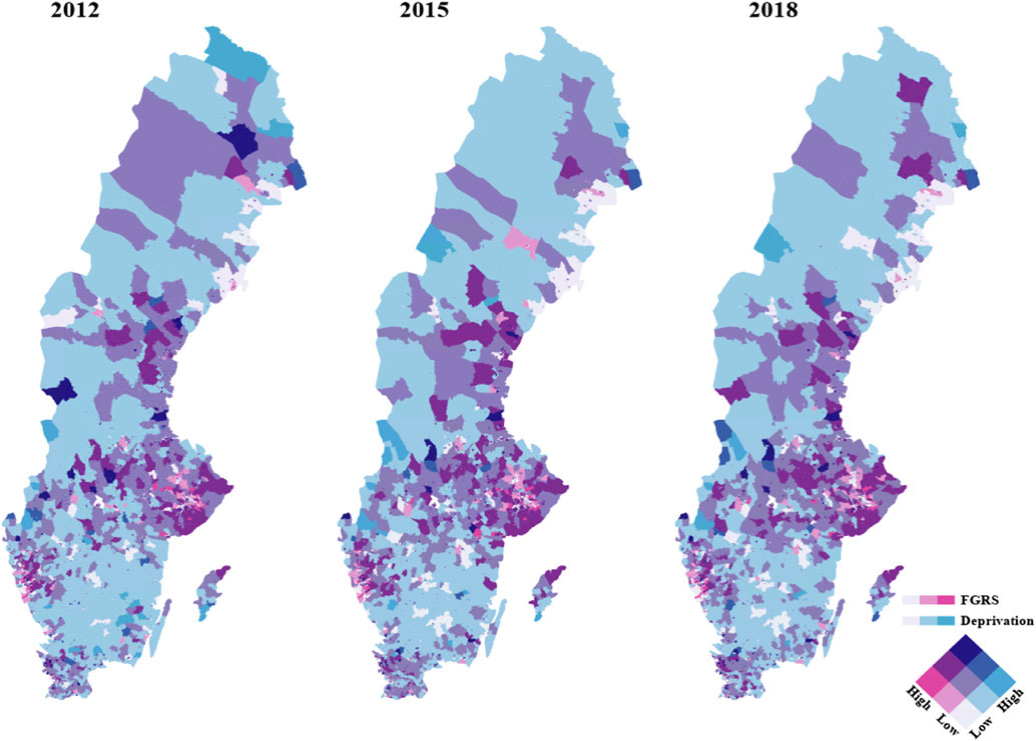
Mean FGRS_DUD_ and Social Deprivation By DeSO in the Country of Sweden in 2012, 2015 and 2018. For the coding of level of FGRS_DUD_ and Social Deprivation by DeSO in this figure, see the legend of Fig. 4.

Finally, we examined the temporal stability of our FGRS_DUD_ and s.d. over the years 2012, 2015 and 2018. Across all DESOs, the cross-time correlations for the FGRS_DUD_ were quite high, equaling +0.90 for 2012 to 2015, +0.91 for 2015–2018 and 0.86 for 2012–2018. For s.d., the parallel figures were slightly higher: +0.97 across all comparisons. The correlation across all DESOs of the FGRS_DUD_ and s.d. measures were also relatively stable equaling +0.62 in 2012, +0.62 in 2015 and, as noted above, +0.63 in 2018. In Fig. 5, the temporal stability of FGRS_DUD_ and s.d. by DeSO is seen for 2012, 2015 and 2018. The maps for the FGRS_DUD_ alone over those years are seen in Appendix Fig. 1. While the general pattern of findings is relatively constant across this 6 year period, modest shifts are seen in a number of specific regions. The correlations for the DESO similarities, as classified by both FGRS_DUD_ and s.d. across time, were, however, very high: 2012–2015: +0.97, 2012–2018: +0.97 and 2015–2018: +0.99 strongly indicating that a high degree of temporal stability exists for both factors.

## Discussion

Before we discuss the three questions we examined in this paper, we provide a summary of the social geography of Sweden that is a helpful background for the interpretation of our findings for readers unfamiliar with this topic.

Sweden's current socio-spatial structure can be seen to be influenced by three main factors: (i) A metropolitan – non-metropolitan gradient (Malmberg & Clark, 2021); (ii) Within metropolitan differentiation in the form of socioeconomic segregation (Haandrikman, Costa, Malmberg, Rogne, & Sleutjes, 2023; Malmberg, Andersson, Nielsen, & Haandrikman, 2018) and (iii) Regional differentiation based on historical patterns of economic specialization (Jansson, Wastenson, Aspenberg, & Tanner, 2011; Wastenson & Alvstam, 1995). The metropolitan non-metropolitan gradient can be found in most developed countries and, according to many observers, has been aggravated in recent decades. It is sometimes reflected in variation in educational attainment, income, ethnicity, political beliefs, and moral values (Kawalerowicz & Malmberg, 2021). The third factor reflects patterns of regional specialization that go back to the 19th century which include differences in natural resource endowments, agricultural conditions, and location relative to markets and possibilities for sea transport. These geographical factors have, in turn, shaped local economic development, class structure, patterns of inter-regional migration, and local cultural development (Mellander & Florida, 2007).

Based on these three factors, what are the most important geographical patterns in Sweden that can have influenced key risk factors for DUD?

### The metropolitan/nonmetropolitan gradient

Sweden has three metropolitan areas: Stockholm (population 2.4 million), Gothenburg (population 1.1 million), and Malmö (population 0.8 million) (Sweden, 2023). Each consists of a metropolitan core and surrounding suburbs. Stockholm is at the center of Sweden's most densely populated area, Middle Sweden, with a number of larger cities (Uppsala, Västerås, Gävle, Örebro, Linköping, and others) that are inter-connected through fast railways and freeways. Gothenburg is the regional center of West Sweden, with a more restricted influence zone (Borås, Trollhättan, Skövde, Varberg). Malmö, connected by a bridge to Copenhagen, is integrated with the larger Copenhagen region, but also serves as a regional node for much of southern Sweden (Lund, Helsingborg, Kristianstad).

Nonmetropolitan Sweden consists of municipalities at different levels of the urban hierarchy: Large cities, some of which are important regional centers, especially in Northern Sweden (Sundsvall, Östersund, Umeå, Skellefteå, Luleå, and others), medium sized cities, smaller cities, and rural areas. Rural areas, far from regional centers or metropolitan areas, dominated by forests and with low population density, are mainly found in Northern Sweden's inland, and further south along the border to Norway (Jansson et al., 2011). There are also forested areas in Middle and Southern Sweden, but these areas have a higher population density (Wastenson, Öberg, & Springfeldt, 1991).

Besides being the largest metropolis, Stockholm has the highest level of socioeconomic segregation. In contrast with metropolitan areas in the US, residential areas with high concentrations of immigrants and often with high levels of s.d. are not in the metropolitan core but rather in relatively distant suburbs which are interspersed with suburbs dominated by middle class residents. The most affluent areas are found in locations close to the Stockholm archipelago or to Lake Mälaren as well as in the inner city (Haandrikman et al., 2023).

A similar structure is found in Gothenburg: A gentrified inner city, rental apartment building suburbs with a high proportion of low-income migrants, single family housing middle class suburbs, and affluent areas with close access to coast. In comparison, the gentrified areas of Malmö are smaller. Malmö also has a large area with a high concentration of low-income migrants that is located close to the city center and is surrounded by a ring of middle class communities strewn across the fertile plains of Sweden's most important agricultural region.

### Regional differentiations

Being elongated along a north to south axis, Sweden is characterized by large climactic differences which results in poor agricultural conditions in the extreme north compared to some south regions where the conditions are excellent. As a heritage from the latest glaciation, when almost the whole of Sweden was covered by ice, much of Sweden is covered by coarse moraine, which can hinder agriculture, except where processes of sedimentation have produced better soil types. As a result, Sweden has extensive forests that became a central resource during Sweden's industrialization. Another important resource is iron ore found in many places in central Sweden. A high dependence on natural resources was a factor that stimulated a spatially dispersed pattern of industrialization with many smaller and medium sized industrial towns being established outside the metropolitan regions (Wastenson & Alvstam, 1995). During the last decades of the 20th century employment in these industries declined sharply producing a large number of towns that no longer have a strong manufacturing sector and that have experienced population decline (Lundmark, 2010). More recently, many such towns have experienced a population turn-around as they have become destinations for Sweden's growing migrant population. These former manufacturing areas have fewer active church goers than the more conservative non-metropolitan areas (Jonsson, Svensson, & Sandberg, 2020).

### The spatial distribution of genetic risk for SUD

To address the first of the three questions we posed, we explored the spatial distribution, within the country of Sweden, of the mean genetic risk for DUD per DeSO in 2018. The distribution is substantially heterogeneous both within the country as a whole, as well as in each of its urban centers. This impression is supported by hot-spot analyses that documents a substantial number of statistically significant hot and cold spots across the country and within urban centers.

Overall, as seen in Fig. 1, on the national scale, the FGRS_DUD_ shows a spatial distribution with high levels of risk centered on the three metropolitan areas of Sweden and there hinterlands, with Stockholm having by far the largest hinterland, followed by Gothenburg, and Malmö, that has only a small hinterland with elevated risks. The Stockholm hinterland encompasses much of populated central Sweden with a number of relatively large cities as well as smaller cities and towns that, traditionally, have been Sweden's manufacturing belt. This also includes the coastal area of lower Northern Sweden, with a historical concentration of forest industries.

Within each of the major metropolitan areas, the highest risk levels are found in areas characterized by relatively high levels of concentrated poverty, typically located in suburbs with high concentrations of rental housing (Kawalerowicz & Malmberg, 2021). Much of the same pattern also shows up in the hotspots.

With respect to the cold-spots showing significantly lower levels of the FGRS_DUD_, on the national level, these show up in the more peripheral parts of Sweden, in the southeast, along the border with Norway, and in parts of Northern Sweden. The distribution of the coldspots is positively correlated with the map of the proportion of residents that voted for the Swedish Christian Democrats which, in turn, is a recognized indicator of high rates of membership in evangelical churches.

### Co-localization of genetic risk for drug use disorder and social deprivation

Our county-wide correlational analysis (Fig. 3) showed a considerable but far from perfect association across all DeSOs in Sweden between the mean FGRS_DUD_ and level of s.d. as characterized by a widely used and well validated measure (Winkleby et al., 2007). We then explored these associations by jointly mapping both variables (Fig. 4). The DeSOs characterized by both high FGRS_DUD_ and s.d. levels (dark blue in Fig. 4) were typically seen in metropolitan locations characterized by concentrated poverty discussed above. Areas that had high genetic risk in combination with low levels of s.d. (red and purple) where more typically seen in the metropolitan hinterlands. Thus, there is a more pronounced joint gradient in s.d. and genetic risk in metropolitan areas, possibly because of selective migration. It could also be that the s.d. index is better at capturing social vulnerability in metropolitan context than in less densely settled areas.

As demonstrated in a recent paper, substantial concentrations of vulnerable individuals can be found outside the metropolitan areas even though the level of deprivation on the DESO level is moderate (Andersson & Borg, 2023). One example given in that study is the high proportion of distressed singles in more peripheral areas. Another example is distressed couples in single family housing living outside the metropolitan core areas. In both cases, there is some overlap with pink areas, that is low s.d. in combination with elevated FGRS_DUD_.

Finally, those areas with low to moderate levels of deprivation in combination with low FGRS_DUD_ risks (light blue) were mostly located in peripheral areas outside of the metropolitan hinterlands. Many of these areas are also characterized as being a part of the Swedish Bible-belt, as indicated, recently, by high levels of votes for the Swedish Christian Democrats.

### Temporal stability of our findings

Third and finally, we examined the temporal stability of the patterns that we observed in 2018. All of them were high suggesting that the factors influencing the localization of genetic risk for DUD and its substantial but far from complete degree of colocalization with s.d. in Sweden were not highly transient. A possible tendency is that the higher risk area in the hinterland of Stockholm has expanded somewhat in a westerly direction from 2012 to 2018. There also seem to be some expansion of the high risk area of South Norrland.

### Context of our findings

We know of no prior examination of the geographical distribution of genetic risk for DUD with which to compare our findings and broadly similar prior studies are rare. Among the most relevant are two studies by Abdellaoui et al., based on UK Biobank data from 2019 (Abdellaoui et al., 2019) and 2022 (Abdellaoui, Dolan, Verweij, & Nivard, 2022). The first examined the geographical distribution of genetic risk for low educational attainment and SES (assessed by the Townsend index [Townsend, Phillimore, & Beattie, 1988]) in the United Kingdom from polygenic scores obtained from the UK Biobank (Abdellaoui et al., 2019). They concluded that the geographical clustering of these measures of what they termed ‘social stratification’ have likely arisen from internal migration driven by socio-economic forces. In the second paper, they expand on the role of internal migration focusing on active gene-environmental correlation (Knafo & Jaffee, 2013), in which genetic liabilities to a range of social and psychological traits influence the selection by individuals of places of residence and the associated social environments. One of these factors they consider is substance use (Abdellaoui et al., 2022). A third study of interest involves mapping of genetic and environmental risk for schizophrenia in Denmark, examining both their main effects and their interaction (Fan et al., 2018).

This study is a descriptive one, moving beyond our prior effort to map the geography of rates of DUD in Sweden (Stewart et al., 2023), focusing on the spatial distribution of two well recognized DUD risk factors. We would suggest the following major conclusions. First, as expected, the geographical distribution of the mean genetic liability to DUD in DeSOs in Sweden is substantially but far from completely correlated with the level of s.d. Second, levels of FGRS_DUD_ tend to be higher in the peripheral parts of the metropolitan core. This could be a result of families with a high genetic risk for DUD being disadvantaged in the labor market and, thus, are less able to get housing where house prices are elevated. This hypothesis is supported by the evidence that genetic risk for DUD is correlated with low educational attainment (Kendler et al., 2021a, 2021b). Third, a trend is seen for the FGRS_DUD_ to be elevated in former manufacturing areas that have experienced industrial decline. One explanation for this could be selective outmigration from these depressed areas of individuals with high educational aspirations, with education having a protective effect against DUD. This has some potential parallels to the geographical distribution of DUD and particularly opiate drug use disorder in the US (Cicero, Ellis, Surratt, & Kurtz, 2014). Finally, we see, in Sweden, areas with a high proportion of active Christians having lower genetic risk for DUD. A large literature supports the protective effect of religious involvement on rates of DUD (Gartner, Larson, & Allen, 1991; Kendler, Gardner, & Prescott, 1997; Koenig, McCullough, & Larson, 2001; Vance, Maes, & Kendler, 2010) but these results at least suggest that some of that association may be mediated by genetic factors perhaps resulting from differential migration into or out of such regions. It is also likely that social effects at a community level – reduced tolerance for drug use or drug dealing – could also mediate this effect.

### Limitations

These results should be interpreted in the context of five potential methodological limitations. First, the assessment of DUD by registry data cannot replicate assessment by personal interview. This approach has obvious advantages, as there are no refusals and the concerns about accurate reporting for behaviors that could be seen as social undesirable are not relevant. Furthermore, genetic epidemiological findings for substance use disorders in Sweden are similar to those found in other samples (Kendler et al., 2012; Kendler et al., 2015; Kendler et al., 2016; Kendler, Maes, Sundquist, Ohlsson, & Sundquist, 2013). However, we will miss individuals whose drug abuse does not lead to medical attention or are associated with criminal behaviors, suggesting that we are, on average, detecting a more clinically severely ill group than would be seen with epidemiological samples assessed by interview.

Second, the FGRS is an index of risk based on the phenotypes of members of the extended family and are not directly comparable to polygenic risks scores obtained from molecular genetic studies. The FGRS has performed well in a number of empirical applications with simulations and comparisons with other similar methods (Hujoel, Gazal, Loh, Patterson, & Price, 2020), suggesting that is provides a good measure of genetic risk (Kendler et al., 2021a, 2021b, 2023a, 2023b, 2023c, 2023d, 2023e). Furthermore, recent empirical comparisons for polygenic risk scores and related simulation studies, suggest that both FGRS-like phenotypic measures and polygenic risk scores, with available samples, are best seen as modestly accurate measures of the same underlying genetic susceptibility (Krebs et al., 2023). Furthermore, our sample was restricted to those individuals that were born in Sweden with two Swedish-born parents because immigrants have too few relatives in the registries to develop meaningful FGRS. Thus, we cannot generalize our families to immigrant native groups in Sweden.

Third, could our results be confounded by a robust effect of s.d. on our ascertainment of DUD and hence our FRGS_DUD_? Given the wide accessibility of health care in Sweden, we considered this hypothesis implausible but we tested it empirically by comparing the s.d.-FRGS_DUD_ association in DeSO areas where we divided our DUD cases into those ascertained by the medical *v.* criminal registries which would likely be subject to quite different potential SES biases. In 2018, the correlation between our DeSO measures of s.d. and FRGS_DUD_ equaled +0.60 when we ascertained our cases of DUD using the medical registry and +0.61 when we did a parallel calculation using DUD ascertained via the criminal registries. We conclude that s.d. related biases in our DUD ascertainment is unlikely to substantially major influence our findings.

Fourth, the major focus of our study is across the temporal window from 2012 to 2018. To address qualitatively the question of the overall temporal stability of our findings, we present in Appendix Fig. 2, a map formatted exactly the same way for Fig. 4 from 2018 done in the Swedish population in 2000. While different in many specifics, the general pattern of findings is broadly stable time period.

## Conclusions

The geographical distribution of the genetic risk to DUD is quite variable in Sweden with statistically defined hot and cold spots. Hot spots were commonly seen in urban areas and those with high levels of social deprivation. Cold spots were most commonly associated with rural areas and areas with high levels of religious affiliation. Examined country-wide, levels of s.d. and FRGS_DUD_ were substantially correlated, but were disassociated in a number of regions. We hope this effort lays the groundwork for further studies of the sources of geographic variation in rates of DUD in Sweden including how such patterns arose historically and how genetic and environmental risk factors have contributed to these patterns.

## Supporting information

Kendler et al. supplementary material Kendler et al. supplementary material

## Supplementary material

The supplementary material for this article can be found at https://doi.org/10.1017/S0033291724002745
