# Supplementary material for: The geographical distribution of the family-genetic risk score for drug use disorder in Sweden and its co-localization with areas of social deprivation
Source: Psychol Med. 2024 Dec 2;54(15):4419–28. doi: 10.1017/S0033291724002745 (PMC11650187; doi:10.1017/S0033291724002745)

APPENDIX

**Table 1 - Definition of Drug Use Disorder**

|  | Registers Used | Definition |
| --- | --- | --- |
| Drug Use Disorder (DUD) | The Swedish Hospital Discharge Register (coverage 1973-2018); Outpatient Care Register (national coverage 2001-2018); Primary Care Registry (Partly coverage from 1999-2018); the Swedish Drug Register (2005-2018); the Swedish Mortality Register, and the Swedish Criminal Register (1973-2018) and the Swedish Suspicion Register (1998-2018) | Drug abuse (DA) was identified in the Swedish medical and mortality registries by ICD codes (ICD9: Drug psychoses (292) and Drug dependence (304); ICD10: Mental and behavioral disorders due to psychoactive substance use (F10-F19), except those due to alcohol (F10) or tobacco (F17)); in the Suspicion Register by codes 3070, 5010, 5011, and 5012, that reflect crimes related to DA; and in the Crime Register by references to laws covering narcotics (law 1968:64, paragraph 1, point 6) and drug-related driving offences (law 1951:649, paragraph 4, subsection 2 and paragraph 4A, subsection 2). DA was identified in individuals (excluding those suffering from cancer) in the Prescribed Drug Register who had retrieved (in average) more than four defined daily doses a day for 12 months from either of Hypnotics and Sedatives (Anatomical Therapeutic Chemical (ATC) Classification System N05C and N05BA) or Opioids (ATC: N02A). |

**Table 2 - Calculation of the Familial Genetic Risk Score (FGRS)**

|  |
| --- |
| The dataset for the calculations includes:  Column1 = Identification number of the proband (Born 1960-1995)  Column2 = Identification number of the relative (1st to 5th degree relatives)  Column3 = Proportion of shared additive genetic effects (0.03125 to 0.50) with the proband  Column4 = Year of Birth of relative  Column5 = Sex of relative  Column6 = Age at registration for trait  Column7 = Age at end of follow-up (2018-12-31 or age at death, or age at emigration whichever came first) |
| **Step 1:** Using all unique relatives with a registration for the disorder, we non-parametrically estimated the distribution of Age at first registration. The empirical distribution is used to obtain weights for relatives without a registration for the disorder, in order to account for the proportion of the time-at-risk period they had completed at the end of follow-up. For example, for relatives at age x at end of follow-up, the weight corresponds to the proportion of relatives registered for the trait that had been registration at age x. For relatives born prior to 1958 we subtracted age at the end of follow-up with the following formula: 1958 - Year of birth of relative. This modification was done in order to control for registration effects (i.e, most registers in Sweden start in 1973 suggesting that relatives from early birth cohorts do not have the possibility to be registered at younger ages). Note that all relatives with the disorder are weighted one. |
| **Step 2**: Transform the binary variable (trait yes/no) into a z-score based on the threshold for each trait. The underlying liability of the individual is not assessable. Instead we estimated the mean of the underlying liability to obtain sex and birth decade specific Z-scores for relatives with the trait registration and relatives without the trait. We generate n random numbers from a N(0, 1) distribution and estimate the mean for relatives registered with the disorder (i.e., mean of the observations above the threshold) and for relatives without a registration (i.e., mean of all observation below the threshold). The thresholds are calculated for each decade of birth and sex. |
| **Step 3**: Correct for cohabitation effects. To estimate the cohabitation effect (i.e. “shared environment”), we created a database with all individuals in the Swedish population born in Sweden 1955-1990. We also included the number of years, during ages 0-15, that individuals resided in the same household as their biological father. We thereby were able to define two kinds of families i) “not-lived-with” father families (offspring never resided for more than 1 year in the same household or in the same community as their biological father); ii) “lived-with” father (offspring resided a minimum of 13 year in the same household as their biological father. We performed a logistic regression model with the binary trait in offspring as outcome and the binary trait in father, type of father, and their interaction as predictors. We used the interaction term as the difference of effect between genes only and genes + environment. The same approach was performed for half-siblings where we compared those who were reared together versus reared apart. The following interaction terms were used in the calculations for each of our disorders:   \|  \| Parent/Children \| Siblings \| \| --- \| --- \| --- \| \| DUD \| 0.92 \| 0.52 \| |
| **Step 4**: Calculate the product for each relative using the four components:   1. Z-score (reflecting sex and year of birth adjusted rates) 2. Weight (reflecting the proportion of risk period they had completed) 3. Cohabitation effects 4. Proportion of shared genetic effects (0.03125 – 0.5) with the proband |
| **Step 5**: Average the product calculated in step 4 across all relatives to a proband |
| **Step 6**: Correct for the number of relatives. We multiplied the results from step 5 with a shrinkage factor. Shrinkage factor (SF): B/(B+A/C). It produces more shrinkage if B and C are small and A is large.   1. the variance of the z-score of the disorder across all relatives, 2. the variance in the mean z-score across all probands, 3. the weighted number of relatives for each proband (sum of Column 3 across each proband). |
| **Step 7**: Correct for difference by year of birth and county differences. There are 21 counties in Sweden. For each proband we used the county they had resided in during the maximum number of years (measured from 1969 and onwards) We standardized the risk score by year of birth and county of the proband into a z-score with mean 0 and SD 1. |

Table 3 – Number of DeSOs in Sweden’s three major urban areas contain DESOs with relatively high and low levels of mean genetic risk for DUD.

|  | Number of DeSOs | Very Low | Low | Medium | High | Very High |
| --- | --- | --- | --- | --- | --- | --- |
| Stockholm | 544 | 117 (21.5%) | 187 (34.4%) | 105 (19.3%) | 86 (15.8%) | 49 (9%) |
| Gothenburg | 306 | 74 (24.2%) | 83 (27.1%) | 60 (19.6%) | 53 (17.3%) | 36 (11.8%) |
| Malmo | 191 | 25 (13.1%) | 65 (34.0%) | 51 (26.7%) | 36 (18.8%) | 14 (7.4%) |

Figure 1 - FGRS_DUD_ by Desos in Sweden 2012, 2015 and 2018.


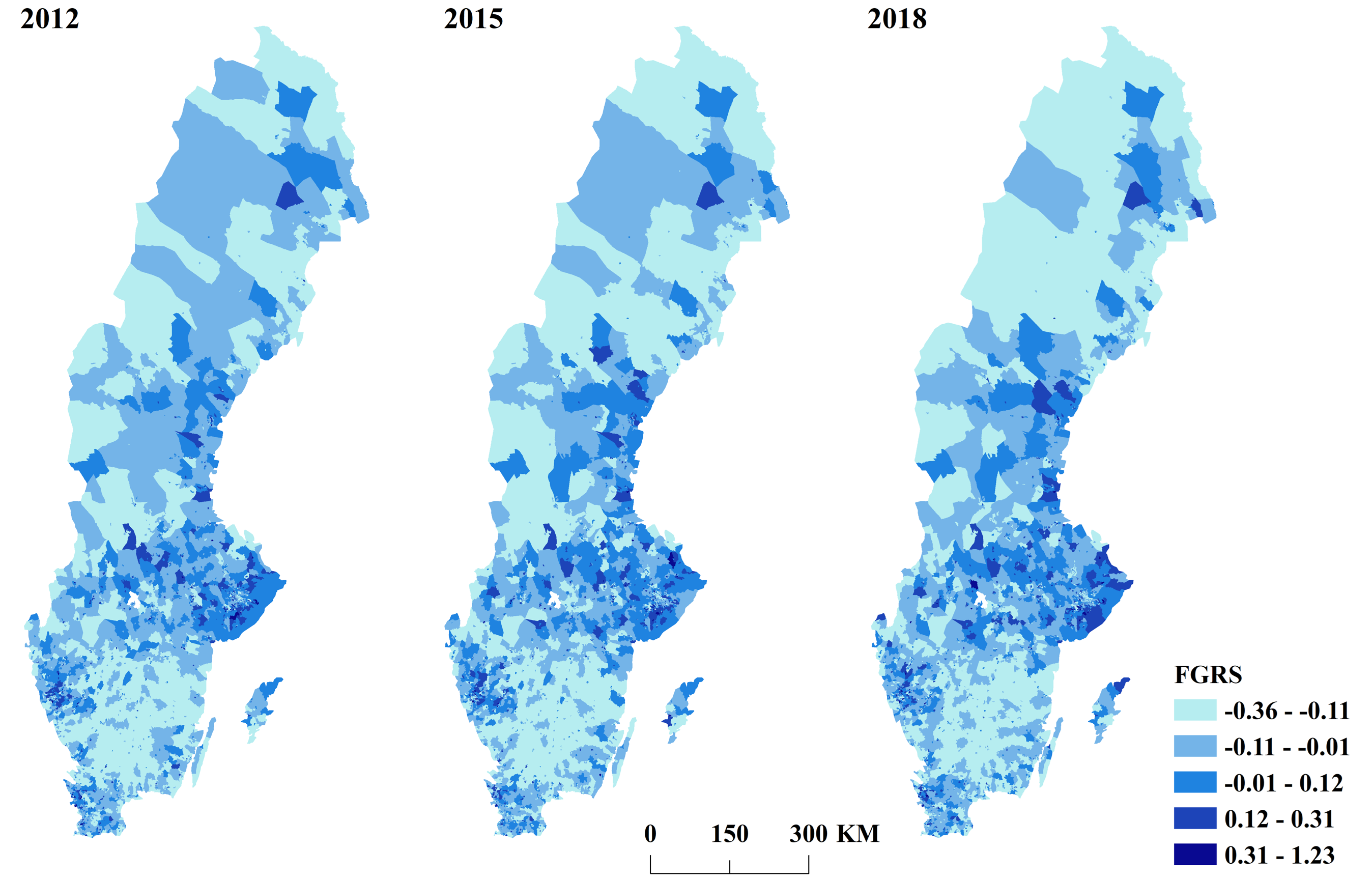


Figure 2 – Maps formatted in exactly the same way as figure 4 from 2018 in the main manuscript performed in the Swedish population in the year 2000


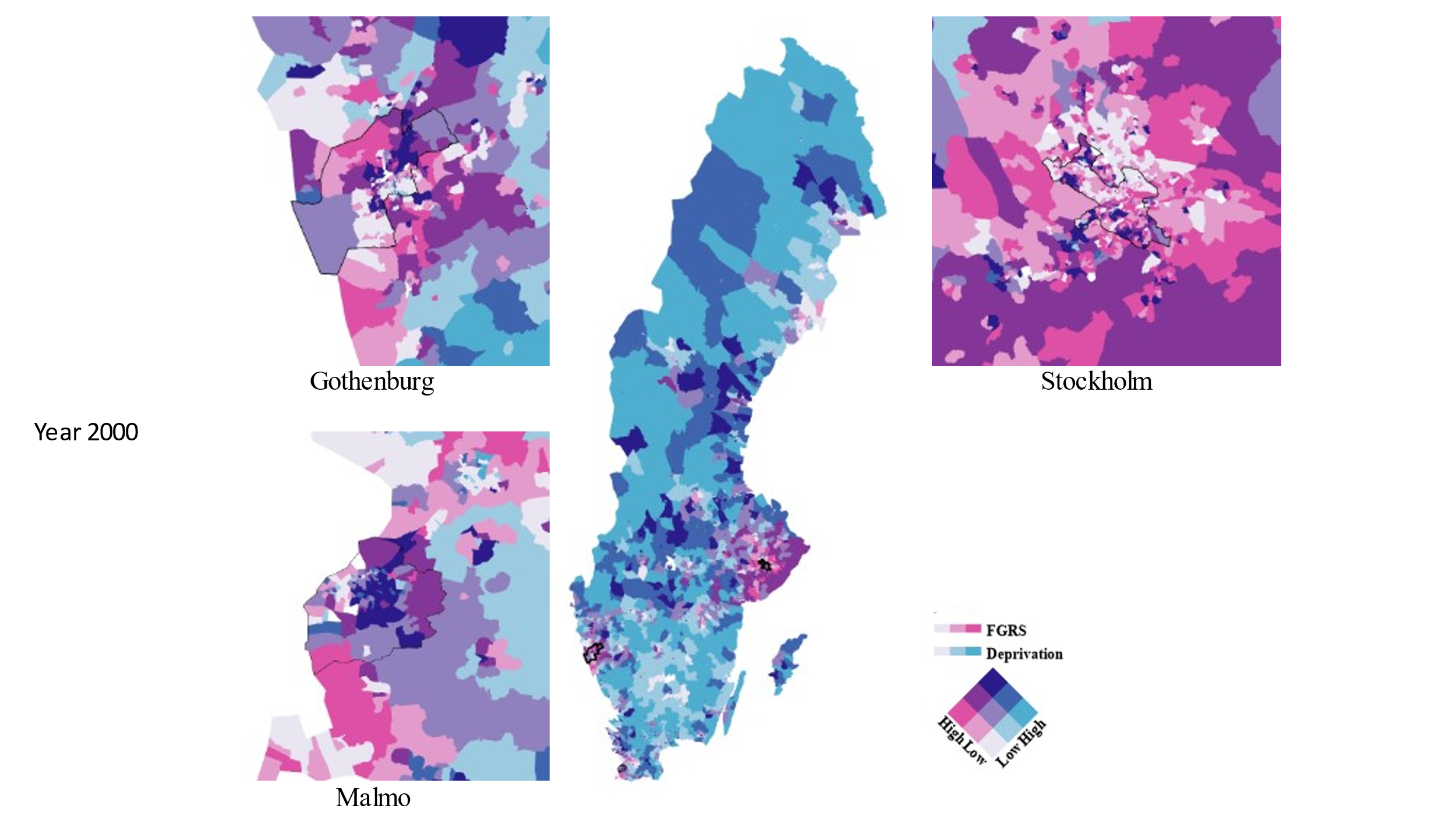

Supplement: Kendler et al. supplementary material [file S0033291724002745sup001.docx]
